# Pandemic preparedness and management in European out-of-hours primary care services – a descriptive study

**DOI:** 10.1186/s12913-023-09059-6

**Published:** 2023-01-19

**Authors:** Ingrid Keilegavlen Rebnord, Guri Rortveit, Linda Huibers, Jonas Nordvik Dale, Marleen Smits, Tone Morken

**Affiliations:** 1 Department of Global Public Health and Primary Care, University of Bergen, Bergen , Norway; 2 National Centre for Emergency Primary Health Care, NORCE Norwegian Research Centre, Bergen, Norway; 3 Research Unit for General Practice, NORCE Norwegian Research Centre, Bergen, Norway; 4 Research Unit for General Practice, Aarhus University, Aarhus, Denmark; 5 Scientific Center for Quality of Healthcare (IQ healthcare), Radboud university medical center, Nijmegen, The Netherlands

**Keywords:** Primary care, COVID-19, Pandemic, Out-of-hours Medical Care, Emergency primary care, Preparedness

## Abstract

**Background:**

Primary care is the first point of contact for all acute health problems. As such, primary care was at the frontline in the COVID-19 pandemic, playing a significant role in clinical responses and information to the public. This study aimed to describe the variations in patient management strategies used in the out-of-hours services in different European countries during the first phase of the pandemic.

**Method:**

We conducted a cross-sectional web-based survey in August 2020, selecting key informants from European countries using European networks. The questionnaire was developed in collaboration with researchers in the field of out-of-hours primary care. We performed descriptive analyses per region, structuring results into themes.

**Results:**

Key informants from 38 regions in 20 European countries responded. Seven regions reported that their out-of-hours services had a pandemic preparedness plan, three had trained on the plan, and two had stockpiles of personal protection equipment before the outbreak. Extension of telephone triage lines and establishment of local infection-control teams and clinics were the main patient management strategies. Other strategies for patient contacts were also used in the regions, such as video-consultations (13 regions), electronic consultations (21 regions), patient’s car as alternative waiting room (19 regions), outside tents for testing (24 regions), “drive-through” testing (26 regions), and separate departments for infected patients (14 regions).

**Conclusion:**

Few out-of-hours services were well prepared for a pandemic, but all expanded and reorganized rapidly, adopting new strategies for patient management and treatment. The results could be useful for planning of organization preparedness of out-of-hours primary care service for future pandemics.

**Supplementary Information:**

The online version contains supplementary material available at 10.1186/s12913-023-09059-6.

## Background

General practitioners (GPs) are the core of primary care, delivering curative and preventive health care and gatekeeping. GPs provide accessible, continuous, comprehensive, and coordinated patient-focused care [1]. Public health care providers that promote, protect, and improve the health of individuals and communities are also part of primary care, delivering essential public health functions such as infectious disease prevention, vaccination, and surveillance of diseases [2]. In addition, out-of-hours (OOH) primary care services are essential in delivering safe, efficient, and cost-effective health care to the population outside office hours.

Different organizational models for OOH services have been developed based on geography, population density, and organization of daytime primary care [3]. In the last decade, implementation of telephone triage and upscaling and centralizing the organization of OOH care were the most important changes [4]. In many European countries, the GP cooperative is the most frequently used organizational model for OOH primary care service [4]. Other countries handle OOH primary care contacts at hospital-based emergency departments.

Pandemics put a large strain on both primary and secondary health care systems. With a constant capacity, work pressure is influenced by patient demand, which is highly influenced by seasonal variations, epidemics, and pandemics. Whereas seasonal variations can be planned for, adaptation of capacity during pandemics is specifically challenging. The last global test was during the H1N1 influenza pandemic in 2009 [5]. A well-organized primary care is essential to prevent overcrowding of secondary care during a pandemic, by information, triage, testing, follow-up of close contacts, isolation and treatment of infected people, and gatekeeping to secondary care [6, 7].

During the COVID-19 pandemic, primary care services made various adaptations to cope with the pandemic. In some countries, primary care providers rapidly adapted their activities to focus on advising, triaging, and managing treatment of COVID-19 cases [8]. Face-to-face contacts were substituted by telephone and video consultations [9, 10]. However, less is known about the adaptations made by OOH primary care services. One study in Norwegian OOH primary care services reported on the use of various patient management strategies to handle a high patient volume, to organize units to avoid virus transmission to health care workers and other patients, and to implement systems for testing, tracing, and isolation [11]. However, studies into the extent and details of reorganization in OOH primary care services in other European countries during the COVID-19 pandemic are lacking. Gaining more insight into management strategies and adaptations at OOH primary services could be useful for local health authorities in future local, regional, or national/global outbreaks. Therefore, the aim of this study was to identify patient management strategies in OOH primary care services during the first phase of the COVID-19 pandemic and to describe variations between different European countries.

## Method

### Design

We performed a cross-sectional web-based survey among key informants from 30 European countries. The authors designed the questionnaire in Qualtrics software (version 2020 of Qualtrics, copyright© 2020, Provo, UT).

### Study population

We included all 27 European Union (EU) countries, in addition to Norway, United Kingdom, and Switzerland. Key informants with expertise in primary care and organization of OOH services were identified by means of the mailing list used for a previous study of OOH organizational models in Europe [4]. In that study, the national delegates of three international organizations (European research network for out-of-hours primary health care (EurOOHnet), the European Association for Quality in General Practice/Family Medicine (EQuiP), and the World Association of Family Doctors (Wonca)) were asked to participate themselves or provide contact information of other experts. The members of EurOOHnet were especially encouraged to participate [12]. To ensure maximum inclusion of experts with experience in the research area, the 168 national delegates in the original mailing list were asked to provide contact information of experts who could fill in the questionnaire. This snowball effect culminated in 184 potential key informants, who were contacted for participation in the study.

### Questionnaire

The questionnaire was developed in collaboration with EurOOHnet members. Based on expert opinion, themes relevant for mapping of management strategies to the pandemic were defined. Several email feedback rounds were conducted to nine EurOOHnet members to achieve face and content validity. A pilot test was performed, asking 15 Norwegian OOH researchers for feedback on the questionnaire. The final questionnaire included five main themes: (1) Pandemic preparedness, (2) Organization of the telephone service during the first five months of the COVID-19 pandemic, (3) Health personnel and staffing, (4) Infection and follow-up routines, (5) Organization of the COVID-19 triage and testing (Additional file 1).

### Data collection

In August 2020, we sent an e-mail to the 184 potential key informants with information about the study and a separate e-mail with an individual access link to the online questionnaire. The goal was to have all EU countries represented with at least one respondent. Reminders were sent to non-responders after three weeks.

In case of multiple respondents from one country, we confirmed that the respondents represented various organizational regions within the country. One respondent submitted an incomplete questionnaire. As the first half was complete, we did not exclude it from our analyses.

### Analyses

We performed descriptive analyses in SPSS (Statistical Product and Service Solutions, version 27.0.1 for Windows, ©SPSS Inc 1989–2020). Analyses were done by summarizing number of respondents, and comparisons were done per region and country.

## Results

### Characteristics of respondents

Among 184 key informants invited, 38 (21%) responded, representing 38 different regions in 20 different European countries (Table 1).

**Table 1:** Distribution of respondents and organizational model* of out-of-hours services (*n* = 38)

Country	Regions (*n*)	Most-used organizational model
Austria	1	Telephone triage and advice services
Belgium	2	GP cooperatives
Bulgaria	1	Rota groups
Croatia	1	Individual GP practices
Czech republic	1	Integrated primary care in hospitals
Denmark	3	GP cooperatives
Estonia	3	Rota groups and Emergency departments
Greece	3	Primary care centers
Hungary	2	Integrated primary care in hospitals
Ireland	2	GP cooperatives
Italy	3	Rota groups and primary care centers
Latvia	1	Integrated primary care in hospitals
Lithuania	1	Emergency departments
Luxembourg	1	GP cooperatives
Norway	3	GP cooperatives
Portugal	1	Emergency departments
Romania	4	Primary care centers and individual GP practices
Slovenia	1	Primary care centers
Spain	3	Rota groups and primary care centers
Sweden	1	Telephone triage and advice services

^*^We used the same terms for organizational model as in Steeman et al. dividing models into small-scale GP groups, middle-scale, and large-scale organizations [4]. Definitions of the different organizational models are described in Additional file 2

### Theme 1) pandemic preparedness

Seven of the 38 regions reported that the regional OOH service had pandemic plans (Table 2). These regions represented seven countries: Bulgaria, Estonia, Greece, Ireland, Portugal, Slovenia, and Spain. Three regions (in Greece and Spain) reported that the service had trained on pandemics and two regions (in Estonia and Italy) that the service had stockpiles of personal protective equipment (PPE) before the pandemic. Of those with no stockpiles of PPE, only five regions reported that it took less than five days before the equipment was available. A region in Italy reported that it took more than two months to get PPE, and in Romania, it was reported that the shortage was especially huge in primary care.

**Table 2:** Level of pandemic preparedness at out-of-hours services, as answered by respondents from 38 regions

Level of pandemic preparedness	Number of regions (*n*)		
	Yes	No	Not known
Regional OOH service had a pandemic plan before the outbreak	7	20	11
Regional OOH service provided training on pandemics and/or participated in an emergency preparedness training before the outbreak	3	29	6
Regional OOH service had stockpiles of personal protection equipment (facemasks, gloves, glasses, infection coats etc.) intended for an extraordinary situation/pandemic before the outbreak	2	30	6

### Theme 2) Organization of telephone service during the first five months of the COVID-19 pandemic

In 16 countries (covering 33 regions), the government established a national telephone number for general information about COVID-19 to the public (Table 3). National numbers were not established in Spain, Italy, Ireland, and Hungary. However, respondents from regions in Spain, Italy, and Hungary reported that local COVID-19 hotlines were established. In Romania, Portugal, and Norway, both national and local telephone numbers were established.

**Table 3:** Telephone organization during the COVID-19 outbreak, as answered by respondents from 38 regions

Organization of telephone service	Number of regions (*n)*		
	Yes	No	Unknown
Government established a national telephone number for general information about COVID-19 to the public in the period February-June 2020	33	5	0
Local region(s) established a COVID-19 hotline in the period February-June 2020	17	13	8
The regional OOH service expanded its telephone center with extra telephone lines during the pandemic	23	7	8
The capacity of the telephone center of the regional OOH service was large enough to handle all inquiries	15	15	8

### Theme 3) availability of health personnel and staffing

To manage the increased demand, the OOH services hired additional personnel (in 16 regions) or other personnel that normally did not work in the OOH services (in 13 regions). Medical students, volunteers, retired doctors, doctors on leave, researchers, nurses, and ancillary staff were used. Collaboration across different health units was initiated for optimal utilization of personnel.

### Theme 4) infection and follow-up routines

Establishment of local pandemic infection control teams for infection detection and follow-up during the first months was reported in 26 regions within 17 countries (Table 4). In the regions without establishment of such teams, the regular infection-control doctors, GPs, epidemiologists, or national governmental teams were responsible for infection detection and follow-up. Ten regions also used the OOH service for infection detection and follow-up.

**Table 4:** Distribution of out-of-hours services responsibilities, as answered by respondents from 37 regions (*n* = number of regions)

	Total number of regions (*n*)	Establishment of local infection control team in the region (*n*)	OOH service responsible for infection detection and follow up (*n*)	OOH service responsible for follow up of close contacts (*n*)	OOH service responsible for home-based treatment (*n*)
Austria	1	1			
Belgium	2	1			
Bulgaria	1	1			
Croatia	1	1			
Czech republic	1	1			
Denmark	2	2	1		
Estonia	3			1	
Greece	3	1	1	2	2
Hungary	2	1			
Ireland	2				
Italy	3	3			
Latvia	1				
Lithuania	1	1		1	
Luxembourg	1	1			
Norway	3	3	2		1
Portugal	1	1			
Romania	4	4	1	1	1
Slovenia	1	1	1		1
Spain	3	2	2	1	1
Sweden	1	1			
Total	37	26	8	6	6

We also asked about alternative strategies for the assessment and follow-up of patients in the OOH services. Video and electronic consultations were the most used digital contact forms, in addition to physical attendance (Table 5). Additionally, in many regions, patients were asked to wait in their car rather than in a waiting room. Other strategies reported were telephone consultations, a separate pre-triage clinic for infectious patients, and examination in the car.

**Table 5:** Distribution of alternative strategies, as answered by respondents from 37 regions (*n* = number of regions)

	Video consultations (*n*)	Electronic consultations (*n*)	Patient’s own car as waiting room (*n*)	No new strategies (*n*)
Austria		1	1	
Belgium	2		2	
Bulgaria				1
Croatia		1		
Czech republic				1
Denmark	2	1	2	
Estonia	1	2	2	1
Greece		1	1	1
Hungary	1	2		
Ireland	1	2	1	
Italy				2
Latvia		1	1	
Lithuania		1	1	
Luxembourg	1	1		
Norway	1		3	
Portugal	1	1	1	
Romania	1	4	2	
Slovenia		1	1	
Spain	1	1		1
Sweden	1	1	1	
**Total**	**13**	**21**	**19**	**7**

We asked to what extent secondary care (hospitals) gave instructions/procedures to primary care about admission to hospitals during the start of the pandemic. Regional respondents from eleven countries confirmed that they had to follow specific procedures. In Estonia, Norway, Romania, and Slovenia, the OOH doctors were obliged to discuss admissions with the local hospital before referral.

### Theme 5) Organization of the COVID-19 triage and testing

Where and in what way the triage and testing were done varied a lot, also within countries (Table 6). In 11 regions, OOH services performed COVID-19 testing in addition to other regional test possibilities. A separate tent outside (in 24 regions), separate department with “dirty area/entrance” (in 14 regions), and “drive through” (in 26 regions) were the most used methods for testing and triage.

In 34 regions, the staff performing the testing had sufficient personal protection equipment to carry out testing and clinical assessment of potentially infected patients, without endangering their own health. Only Romania and Austria reported such problems, and only for the first two weeks of testing.

**Table 6:** Distribution of responsible units for triage/testing as answered by respondents from 37 regions (*n* = number of regions)

Country	GP practices or regional GP cooperatives (*n*)	OOH-service (*n*)	Local government (*n*)	Hospital (*n*)
Austria	1	1		1
Belgium	2	1	1	
Bulgaria		1		1
Croatia	1		1	
Czech republic	1			
Denmark	1	1	1	2
Estonia	2	2	1	1
Greece				2
Hungary	2			
Ireland	2	1		1
Italy			3	
Latvia	1			1
Lithuania		1		1
Luxembourg	1	1		1
Norway		3	1	2
Portugal			1	
Romania	1	2	3	2
Slovenia	1			
Spain	1	2	2	
Sweden				1
**Total**	**17**	**16**	**14**	**16**

## Discussion

### Main findings

OOH primary care services play a key role in dealing with acute illness in primary health care, but only in very few countries OOH primary care services had a pandemic plan or had done pandemic exercises before the COVID-19 outbreak in March 2020. Most respondents answered that protective equipment became available within weeks, but in some regions it took some months, and they experienced that secondary care was prioritized. Although many similarities existed among countries in the organization of testing, follow-up, and clinical examination, important differences were found. Some national governments build up new teams/offices, while others used the regular primary care system, but added extended telephone service, testing systems, and systems for follow-up of infected and close contacts.

### Strengths and limitations

Although questionnaires have limitations with risk of selection and information bias, we considered this the most feasible method to get a broad picture of many European countries. No validated questionnaire was available. However, the authors who represented a range of countries discussed the questions to ascertain that these were perceived equally, to secure face validity. Still, we cannot rule out that respondents from so many different countries and with different language backgrounds interpreted expressions differently. We used as few answering categories as possible, and we added a free text option to limit this potential bias and secure content validity. The free text answers generally confirmed the answers that were ticked off.

Although the response rate was relatively low, we had answers from 2/3 of the EU countries. As some of the largest European countries, like France, Germany, Great Britain, and the Netherlands, did not participate, the results cannot be generalized to all of Europe. Key contact persons were probably missing for countries with a heterogeneous organization of OOH primary care, more likely larger countries. For countries with heterogeneous organizations, the invited key informants may have felt unable to respond, which might partly explain the low response rate [1].

This study dealt with the first five months of the pandemic, describing pandemic preparedness. Changes took place quickly, and better organization in the face of increasing infections may have occurred later, including increased access to testing and better access to protective equipment.

### Comparison with literature

Our study showed that few countries had pandemic plans involving OOH services to such a degree that key informants were aware of them, even though earlier pandemics have made it clear that national and local pandemic plans are necessary to prevent, control, and respond to viruses with pandemic potential [13]. Primary care is central in all phases, and strengthening, integrating, and defining their role is essential in the preparedness [14, 15].

Very few regions reported that they had stockpiles of protective equipment. Nevertheless, only two countries responded that it was problematic to obtain enough equipment to protect health personnel in primary care during the first months after the outbreak. This result is in strong contrast to what is reported from low and middle income countries [16] and also in high income countries such as the US [17], where severe shortages in personal protective equipment during the COVID-19 crisis have been reported. The prior level of organization of primary care may be a factor here, which in general is high in European countries compared to low-income countries.

Extra personnel were used extensively, including students, retired, and former health care workers. All available health personnel were engaged to staff new organizations as “pandemic clinics” and test stations, and to replace health personnel in quarantine. Other studies have shown a large extent of charity present in the health personnel population that made it possible to get enough staff [18–20]. At the same time, a decline in the number of other infectious diseases and other emergencies contributed [21].

At the outbreak, alternative strategies for patient contacts became necessary. Our respondents reported that telemedicine in the form of video- and e-consultations was implemented, which is in accordance with other studies [10, 22–25]. In some countries, such contact types were in place prior to the pandemic, whereas other countries were not digitally ready to install this during the outbreak. Telemedicine has been used in primary care and emergency departments for monitoring patients, and in tertiary care and mental health care [26, 27]. Our results indicate that the OOH primary care services in many regions within a range of countries used video- and e-consultations, even though professionals have less prior knowledge about the patient compared to a daytime GP, and a physical examination probably is more often necessary compared with daytime primary care, where a substantial part of the patient population has chronic diseases.

### Implications for practice and future research

As our study focused on the first five months of the pandemic, the results may be useful to compare with the organization of the OOH services in later waves of infection, to get insight into the experiences gained through different stages in the pandemic. Also, it would be interesting to repeat the data collection and ask key informants a second time a few years after beginning of the pandemic about preparedness and established strategies. Furthermore, there is a clear potential for improvement of making and training on preparedness plans that local and central health authorities should take seriously. To what extent OOH primary care was part of the initial response to the outbreak varied due to variations in the organization of OOH primary care. This provides potential for evaluation of the most effective methods and organizations during the pandemic and may inform plans for future pandemics.

The rapid and necessary change in contact forms during this pandemic has shown that telemedicine can be a useful supplement to regular consultations in the future, but it remains to be clarified under what conditions this contact form can replace face-to-face consultations.

Our study did not evaluate the quality of pandemic management. However, other research showed that countries with a fragmented health service seem to have suffered largely [7, 28]. Countries that previously had a well-organized primary health service were able to use this to expand capacity, handle triage, testing and follow-up, and screen patients without the need for hospitalization [10, 29]. Yet, there is a great learning potential for future pandemics.

## Conclusion

In only a few regions in European countries, the OOH primary care system was well prepared for a pandemic, but reorganization went quickly during the first months after the outbreak. The capacity in OOH services was extended with telephone lines, extra personnel, and new consultation strategies to both handle large numbers of patients and at the same time protect patients and health care workers from infections. More studies from later phases of the pandemic should be conducted to clarify which strategies are most sustainable over time.

## Supplementary Information

**Additional file 1.**

**Additional file 2.**

## Data Availability

The datasets generated and analyzed during the current study are available from the corresponding author on reasonable request.

## Abbreviations

GP: General practitioner
OOH: Out-of-hours
PPE: Personal protective equipment

## Declarations

The participants provided informed consent for the present study. All methods were carried out in accordance with relevant guidelines and regulations as given by the Declaration of Helsinki.
