# Supplementary material for: Pandemic preparedness and management in European out-of-hours primary care services – a descriptive study
Source: BMC Health Serv Res. 2023 Jan 19;23:54. doi: 10.1186/s12913-023-09059-6 (PMC9849833; doi:10.1186/s12913-023-09059-6)
Supplement: Supplementary file 1 — Additional file 1. [file 12913_2023_9059_MOESM1_ESM.docx]

Additional file 1

EUROOHNET COVID-19

Q1 **The role of out-of-hours (OOH) services during the COVID-19 outbreak.**

Q3 **Background Information**

Q4 Which country do you live and work in?

___________________________________________________________________________

Q5 Which region do you work in?

Q6 How many inhabitants are living in your region?

Q7 Select the most typical organizational model of the OOH service in your region

- Individual GP practices: The GPs take care of their own patients 24/7. (1)
- Rota groups: Small-scale GP groups of about 4-15 members working in the same region. (2)
- GP cooperatives: Large-scale organizations of about 15 to more than 250 GPs. GPs take turns being on duty during out-of-hours, for the patient population of all participating GPs. GP cooperatives may be supported by nurses, management, drivers etc. (3)
- Emergency departments: The GP has no role in the care for patients during out-of-hours; Instead the emergency department of hospitals take care of primary care patients during out-of-hours. (4)
- Integrated primary care in hospitals: GP working at the emergency department of the hospital during out-of-hours. (5)
- Deputizing services: Commercial agencies that employ GPs to take over duties of other GPs. (6)
- Primary care centers: Centers that patient can visit without an appointment, for minor injuries or illnesses. Healthcare professionals in such centers operate under supervision of a GP. (7)
- Minor injury centers or walk-in-centers: Centers, which patients can visit without an appointment for minor injuries or illnesses, to ask a trained nurse for health information, advice and treatment. (8)
- Telephone triage and advice services: Patients can contact a medically trained professional via a national/regional telephone number. This professional gives advice or refers the patient to the most suitable professional. If telephone triage is implemented in another organizational model, this category is not applicable. (9)

Telephone triage during the outbreak Organization of the telephone service Q8 Organization of the telephone service during the first 5 month of the COVID-19 outbreak (February– June 2020)

Q9 Did the government establish a national telephone number for general information about COVID-19 to the public in the period February-June 2020?

- Yes (1)
- No (2)

Q11 Did any local region(s) establish a COVID-19 hotline in the period February-June 2020?

- Yes (1)
- No (2)
- I do not know (4)

Display This Question:

If Did any local region(s) establish a COVID-19 hotline in the period February-June 2020? = Yes

Q12 Who staffed the local number for information about COVID-19?

- Nurses (1)
- Other, describe (2) _______________________________________________

Q13 The regular telephone-center of the OOH-service in your region:  Was the capacity of the telephone-center large enough to handle all inquiries?

- Yes (1)
- No (2)
- I do not know (3)

Q14 Did the telephone-center of the OOH-service in your region expand with extra telephone lines during the pandemic?

- Yes (1)
- No (2)
- I do not know (3)

Display This Question:

If Did the telephone-center of the OOH-service in your region expand with extra telephone lines duri... = Yes

Q15 What kind of personnel handled the extra inquiries during the outbreak on the telephone to the OOH services?

- Nurses/assistants normally staffed at the OOH services (1)
- Other nurses/assistants who were added during the pandemic to handle telephone calls (2)
- Other personnel, describe: (3) ____________________________________________

Q16 **Pandemic preparedness**

Q17 Did the OOH-service in your region have a pandemic plan before the outbreak?

- Yes (1)
- No (2)
- I do not know (3)

Display This Question:

If Did the OOH-service in your region have a pandemic plan before the outbreak? = Yes

Q18 Was the pandemic plan adequate for the measures needed to be implemented in connection with the outbreak?

- Yes (1)
- No (2)
- Partially, describe (3) ________________________________________________

Q19 Had the OOH-service in your region provided training on pandemics and/or participated in an emergency preparedness training before the outbreak?

- Yes (1)
- No (2)
- I do not know (5)

Q20 Did the OOH-service in your region have stockpiles of personal protection equipment (facemasks, gloves, glasses, infection coats etc.) intended for an extraordinary situation / pandemic before the outbreak?

- Yes (1)
- No (2)
- I do not know (4)

Display This Question:

If Did the OOH-service in your region have stockpiles of personal protection equipment (facemasks, g... = No

Q21 How many days did it take before the equipment was available?

- <5 days (1)
- >5 days (2)
- Describe (3) ________________________________________________

Q22 **Health personnel and staffing**

Q23 Did the OOH-service in your region use OTHER personnel (other professions etc) than regular personnel during the pandemic?

- Yes, describe (1) ________________________________________________
- No (2)

Q24 Did the OOH-service in your region use MORE personnel than regular during the pandemic?

- Yes, describe (1) ________________________________________________
- No (2)

Q25 Have GPs who do not normally participate in OOH primary care attended an on-call scheme due to the pandemic?

- Yes, describe (1) ________________________________________________
- No (4)

Display This Question:

If Did the OOH-service in your region use OTHER personnel (other professions etc) than regular perso... = Yes, describe

Or Did the OOH-service in your region use MORE personnel than regular during the pandemic? = Yes, describe

Or Have GPs who do not normally participate in OOH primary care attended an on-call scheme due to th... = Yes, describe

Q26 What were the reasons for the OOH-service in your region to obtain other personnel than usual? (more answers possible)

- The regular staff were in quarantine (1)
- Increased workload (overwhelming number of patients compared to normal situation) (2)
- Staffing of a new respiratory clinic / pandemic clinic/ fever clinic (3)
- Staffing of new smaller teams (4)
- Other, describe (5) _______________________________________________

Q27 **Infection and follow-up routines**

Q28 Has the local region created its own infection control team during the outbreak?

- Yes (1)
- No (2)
- I do not know (3)

Q29 Who was responsible for infection detection and follow-up of test responses?

(more answers possible)

- Local established pandemic infection-control team (1)
- OOH-service (2)
- Regular local infection-control doctor (3)
- GP (4)
- Other, describe (5) _______________________________________________

Q30 Who was responsible for follow-up on close contacts? (more answers possible)

- Local established pandemic infection-control team (1)
- OOH-service (2)
- Regular local infection-control doctor (3)
- GP (4)
- Other, describe (5) _______________________________________________

Q31 Who was responsible for follow-up of home-based COVID-19 patient treatment?

(more answers possible)

- Local established pandemic infection-control team (1)
- OOH-service (2)
- Regular local infection-control doctor (3)
- GP (4)
- Other, describe (5) _______________________________________________

Q32 Was a local department with hospital beds to handle COVID-19 patients outside hospital established? (more answers possible)

- Yes, separate departments/rooms at nursing homes are converted to “Corona departments” (1)
- Yes, extra beds in separate departments at nursing homes (6)
- Yes, separate departments/rooms at OOH-clinics are converted to “Corona departments” (7)
- No, only the hospital had beds to handle COVID-19 patients (2)
- Other, describe (8) _______________________________________________

Q33 Has the OOH-service implemented any of the following alternative strategies for the assessment / follow-up of patients? (more answers possible)

- Video consultations (1)
- Electronic consultations or chat or mail (2)
- Use patients´ own car as waiting room outside the clinic (3)
- No new strategies (5)
- Other, describe (4) _______________________________________________

Q34 During the pandemic period, has the OOH-service received guidelines for admission to local hospital with clear instructions for which patients that will benefit from hospital treatment?

- Yes (4)
- No (5)
- Not relevant (6)

Display This Question:

If During the pandemic period, has the OOH-service received guidelines for admission to local hospit... = Yes

Q35 Was the OOH-doctor obliged to discuss admissions with the local hospital?

- Yes (1)
- No (4)
- Not relevant (5)

Q36 Was it necessary to change the emergency procedures (who and how to handle critically ill patients) at the OOH service as a result of the pandemic?

- Yes, describe (1) ________________________________________________
- No (2)
- Not relevant (3)

Q37 **Organization of the COVID-19 triage and testing**

Q38 Who organised testing for COVID-19 suspected patients on primary level in your region? (more answers possible)

- GP offices separately (1)
- Regional GP cooperatives (2)
- OOH-service (3)
- Hospitals (4)
- Local government (5)

Q39 How was the testing performed? (more answers possible)

- A separate tent outside for testing (1)
- A separate department for testing (2)
- “drive through”- testing (3)
- Other, describe (4) _______________________________________________

Q40 Has the staff performing the testing had sufficient personal protection equipment to carry out testing or clinical assessment of potentially infected patients, without endangering their own health?

- Yes (1)
- No, describe (2) ________________________________________________

Q41 Did the OOH-clinic have separate rooms /pathways for patients with suspected infection? (more answers possible)

- Yes, separate receptions (1)
- Yes, special triage rooms (or tents) (2)
- Yes, separate waiting rooms (3)
- Yes, separate treatment rooms (4)
- Other, describe (5) ________________________________________________
- No (6)

Q42 Has a separate “clinic for infections” / “pandemic clinic” / “fever outpatient clinic” been set up in your region?

- Yes (1)
- No (2)
- I do not know (3)

Display This Question:

If Has a separate “clinic for infections” / “pandemic clinic” / “fever outpatient clinic” been set u... = Yes

Q43 Which personnel was working at the separate clinic? (more answers possible)

- Nurses from the OOH-services (1)
- GPs (2)
- Other nurses (3)
- Personnel from other departments in the primary health care system (4)
- Students (5)
- Other, describe (6) ________________________________________________

Q44 Have the GP practices in your region locked their practices for possible infected patients during the outbreak?

- Yes, the patients had to contact separate clinics/teams (1)
- Yes, the patients had to contact the OOH-services (2)
- Yes, but their GP was available for remote consultations (video, telephone, e-mail…) (3)
- No, they handled their possible infected patients as before (4)
- Other, describe (5) ________________________________________________

Q45 **Nursing homes**

Q46  Has there been a COVID-19 outbreak in the nursing homes in your region?

- Yes (1)
- No (2)

Display This Question:

If  Has there been a COVID-19 outbreak in the nursing homes in your region? = Yes

Q47 How long did it take to get the outbreak under control?

- Less than a week (1)
- More than a week but less than a month (2)
- More than a month (3)

Q48 Who provides medical care in the nursing homes in your region? (more answers possible)

- Nursing home physicians, working only in nursing homes (1)
- Nursing home physicians, working also elswhere (2)
- Contractors (3)
- OOH service (4)
- Emergency services (5)
- Other physicians (e.g. psychiatrists, geriatrician) (6)
- I do not know (8)

Q49 **The burden on the health personnel during the pandemic**

Q50 How many GPs in primary care have been infected with COVID-19 in your country, as far as you know?

- Number (1) ________________________________________________
- I do not know (2)

Q51 How many GPs in primary care have died from COVID-19 in your country, as far as you know?

- Number (1) ________________________________________________
- I do not know (2)

Q52 How many nurses in primary care have been infected with COVID-19 in your country, as far as you know?

- Number (1) ________________________________________________
- I do not know (2)

Q53 How many nurses in primary care have died from COVID-19 in your country, as far as you know?

- Number (1) ________________________________________________
- I do not know (2)

Q54 Thank you for answering. Click next to submit the questionnaire.
