# Supplementary material for: Pandemic preparedness and management in European out-of-hours primary care services – a descriptive study
Source: BMC Health Serv Res. 2023 Jan 19;23:54. doi: 10.1186/s12913-023-09059-6 (PMC9849833; doi:10.1186/s12913-023-09059-6)
Supplement: Supplementary file 2 — Additional file 2. [file 12913_2023_9059_MOESM2_ESM.docx]

**Additional file 2**

Organizational models for OOH services

| Name | Description |
| --- | --- |
| Telephone triage and advice services: | Patients can contact a medically trained professional via a national/regional telephone number. This professional gives advice or refers the patient to the most suitable professional. |
| GP cooperatives | Large-scale organizations of about 15 to more than 250 GPs. GPs take turns being on duty during out-of-hours, for the patient population of all participating GPs. GP cooperatives may be supported by nurses, management, drivers etc. |
| Rota groups | Small-scale GP groups of about 4-15 members working in the same region. |
| Individual GP practices | The GPs take care of their own patients 24/7. |
| Integrated primary care in hospitals | GP working at the emergency department of the hospital during out-of-hours. |
| Emergency departments | The GP has no role in the care for patients during out-of-hours; instead, the emergency department of hospitals take care of primary care patients during out-of-hours. |
| Primary care centers | Centers that patient can visit without an appointment, for minor injuries or illnesses. Healthcare professionals in such centers operate under supervision of a GP. |
| Integrated primary care in hospitals | GP working at the emergency department of the hospital during out-of-hours. |
| Minor injury centers or walk-in-centers: | Centers, which patients can visit without an appointment for minor injuries or illnesses, to ask a trained nurse for health information, advice, and treatment. |
